# Supplementary material for: A Global Screen for Assembly State Changes of the Mitotic Proteome by SEC-SWATH-MS
Source: Cell Syst. 2020 Feb 26;10(2):133–155.e6. doi: 10.1016/j.cels.2020.01.001 (PMC7042714; doi:10.1016/j.cels.2020.01.001)

Q5JRA6 | MIA3_HUMAN | MIA3 KIAA0268 TANGO TANGO1 UNQ6077/PRO20088
Monomer MW [kDa]: 213.702 Monomer expected elution fraction: 34

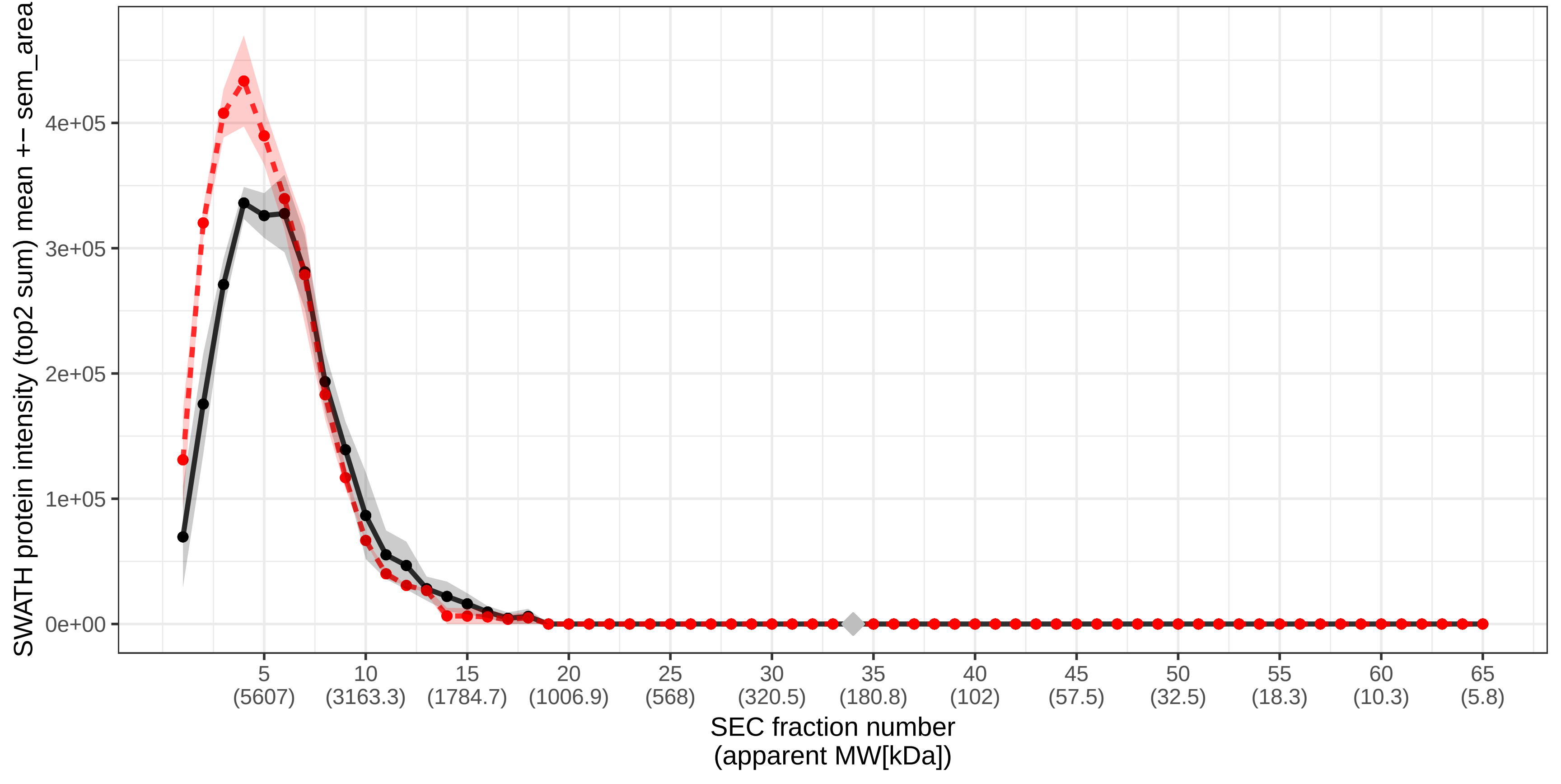

Supplement: Data S1. SEC-SWATH-MS Protein Chromatograms, Related to Figure 1 [file mmc6.zip › SECchrom_Q5JRA6_MIA3_HUMAN_MIA3_KIAA0268_TANGO_TANGO1_UNQ60.pdf]
